# New Insights into the Occurrence of Matrix Metalloproteases -2 and -9 in a Cohort of Breast Cancer Patients and Proteomic Correlations

**DOI:** 10.3390/cells7080089

**Published:** 2018-07-28

**Authors:** Gianluca Di Cara, Maria Rita Marabeti, Rosa Musso, Ignazio Riili, Patrizia Cancemi, Ida Pucci Minafra

**Affiliations:** 1 Centro di Oncobiologia Sperimentale, Università di Palermo, 90146 Palermo, Italy; lucadicar@gmail.com (G.D.C.); marabetimariarita@yahoo.it (M.R.M.); rosi82.m@libero.it (R.M.); 2 La Maddalena Hospital, 90146 Palermo, Italy; urp@lamaddalenanet.it; 3 Dipartimento di Scienze e Tecnologie Biologiche Chimiche e Farmaceutiche, Università di Palermo, 90100 Palermo, Italy

**Keywords:** matrix metalloproteases, breast cancer, proteomics

## Abstract

Matrix metalloproteases (MMPs) are a family of well-known enzymes which operate prevalently in the extracellular domain, where they fulfil the function of remodeling the extracellular matrix (ECM). Within the 26 family members, encoded by 24 genes in humans, MMP-2 and MMP-9 have been regarded as primarily responsible for the basement membrane and peri-cellular ECM rearrangement. In cases of infiltrating carcinomas, which arise from the epithelial tissues of a gland or of an internal organ, a marked alteration of the expression and the activity levels of both MMPs is known to occur. The present investigation represents the continuation and upgrading of our previous studies, now focusing on the occurrence and intensity levels of MMP-2 and -9 and their proteomic correlations in a cohort of 80 breast cancer surgical tissues.

## 1. Introduction

Breast cancer (BC) is one of the most common types of cancer in women, resulting in more than one million cases annually with disparities in incidence and mortality worldwide [1,2]. BC is a heterogeneous disease, both at the inter- and intra-tumoral levels, leading to different prognostic implications: indeed, some carcinoma subtypes have a benign course, while others behave very aggressively and are potentially metastatic, causing treatment failure and probable mortality [3]. Usually, the prognosis for patients becomes worse in cases of late diagnosis [4]. Thus, periodical screening is of great importance for the detection of early-stage tumors. Concurrently, a fundamental goal for both the diagnosis and therapy of patients is to increase the knowledge of cancer biology.

Modern cancer research aims to provide information on the mechanisms leading to cell transformation and tumor progression to be used for the development of new markers and new therapies. Several traditional prognostic markers for breast carcinoma, including tumor size, nodal involvement, histological tumor type, and differentiation, as well as the age of the patients at diagnosis, are currently in use. Furthermore, biological markers which are predictive for treatments, such as the hormone-dependency of the tumor and its HER-2 expression, are of increasing importance [5,6]. However, new markers are still necessary to better identify and to distinguish patients who need the highly intensive treatments from those who could be spared the most toxic treatments.

Metastasis is the leading cause of death in patients with cancer. Thus, a crucial aspect of tumor progression is the ability of cancer cells to cross tissue barriers and spread to distant anatomical sites. The extracellular matrix (ECM) is the first barrier against cell invasion. Therefore, the proteolytic degradation of the ECM is a key aspect of tumor progression. Matrix metalloproteinases (MMPs) are a family of highly homologous, zinc- and calcium-dependent extracellular enzymes [7]. They are classified into five subgroups (collagenases, gelatinases, stromelysins, matrilysins and membrane metalloproteinases) based on substrate specificity, protein domain structure, sequence homology and the ability/inability to be secreted. Therefore, MMPs may play a critical role in the conversion of an “in situ” breast cancer to an invasive lesion. In particular, MMP-2 and -9 can break down several collagenous components of the basement membranes that surround the tissue confines [8,9]. Several studies in the past decade have shown that these enzymes are involved in breast cancer initiation and growth through the interaction with tumor suppressor genes involved in the early stage of tumorigenesis [10]. Several studies correlated increased levels of the gelatinases with higher metastatic spread and reduced survival [11,12].

However, the complex molecular networks that supervise the “entry into play” of these lytic enzymes is not yet fully understood.

The purpose of this study was to examine the activity levels of MMP-2 and MMP-9 in a cohort of 80 surgical samples of breast cancer patients, and to uncover their putative correlations with the proteomic assembly of the same patients.

## 2. Materials and Methods

### 2.1. Patients and Tissue Samples

In this study, we examined the activity levels of gelatinases in tissue samples from 80 breast cancer (BC) patients, diagnosed as ductal infiltrating carcinomas with histological grading G2/G3. No other clinical markers were included in this study, because they were not required for the aim of the present research. The 30 adjacent non-tumor tissues were located at least 5 cm away from the primary tumor.

The study was carried out after fulfilling all required ethical standards with the informed consent of patients. The tissue samples were obtained following surgical interventions at “La Maddalena” Hospital of Palermo and were immediately cryo-preserved at −80 °C until use. All samples were intended to be discarded after the completion of the histopathological examinations.

The patients in this study did not receive any cytotoxic/endocrine treatment prior to surgery.

### 2.2. Tissue Processing

The frozen samples were weighed, cut into small pieces, homogenized in an ice bath with an extracting buffer (50 mM Tris, 0.003% penicillin, 0.005% streptomycin, pH 7.5) and mixed overnight at 4 °C. After incubation, tissue lysates were centrifuged several times at 10,000 rpm for 20 min at 4 °C in order to remove debris. Supernatants containing proteins were collected and dialyzed against Millipore water for 24 h at 4 °C, with several changes. Samples were then freeze-dried and stored at −80 °C. For the analyses, samples were solubilized in 50 mM Tris pH 7.5, and the protein content was quantified by Bradford assay [13].

### 2.3. Gelatin Zymography

Protein samples (18 µg) in Tris pH 7.5 were solubilized in Laemmli sample buffer under non-reducing conditions and loaded onto SDS-PAGE on 7.5% polyacrylamide gels co-polymerized with 0.1% gelatin at 150 V in a Tris-glycine buffer. Purified MMP-2 and MMP-9 [14] were used as references.

After electrophoresis, gels were washed in a washing buffer (2.5% Triton X-100, 50 mM Tris, pH 7.5) for 1 h to remove SDS and allow a partial renaturation of the protein. Gels were then incubated overnight at 37 °C in an incubation buffer (0.15 M NaCl, 10 mM CaCl_2_, 50 mM Tris, pH 7.5) that allows the activation of the metalloproteinases. Subsequently, gels were fixed and stained with 0.2% Coomassie Brilliant Blue R-250 in 40% methanol and 10% acetic acid, and destained in 7% methanol and 5% acetic acid. The location of gelatinolytic activity was detectable as clear bands against the blue background of stained gelatin.

### 2.4. Quantification of Enzymatic Activity

Following zymography, the degree of gelatin digestion was quantified using ImageMaster 2D Platinum software, Version 5.0 (Amersham, Little Chalfont, UK). The image was digitally inverted, so that the integration of bands was reported as positive values. The pixel density was determined after background subtraction and used to calculate the integrated density of a selected band. Values of integrated density with area were reported in volume units, as calculated by the Image Master algorithms.

### 2.5. Two-Dimensional Gel Electrophoresis

The protein extracts were dissolved as reported previously [15]. Protein samples (45 µg for the analytical gels, or 1.5 mg for preparative gels) were rehydrated in a solution containing 8 M urea, 2% CHAPS, 10 mM DTE and 0.5% carrier ampholytes (Resolyte 3.5–10; Amersham, Little Chalfont, UK), and applied to the strips for isoelectrofocusing (IEF) (18 cm long, pH range 3.0–10, Bio-Rad, Hercules, CA, USA). After the IEF, the strips were incubated in a solution composed of 50 mM Tris-HCl pH 6.8, 6 M urea, 0.5% SDS, 30% glycerol, 130 mM DTE and 135 mM iodoacetamide (Sigma-Aldrich, St. Louis, MO, USA).

The strips containing the focused proteins were then loaded onto 9–16% linear gradient polyacrylamide gels (SDS-PAGE) and the electrophoresis run was performed by applying a constant current of 20 mA/gel at 10 °C. The protein spots were revealed by ammoniacal silver staining [14]. Silver-stained gels were analyzed with ImageMaster 2D Platinum software with the support of the ExPaSy molecular biology server.

### 2.6. Protein Identification and Functional Association

The spots of interest were submitted to peptide mass fingerprinting using the Voyager DE-PRO mass spectrometer (MALDI-TOF, AbSciex, Framingham, MA, USA) as described [16]. The in-gel digestion of the selected protein spots was performed with sequencing-grade trypsin (Promega, Madison, WI, USA). The peptides were re-dissolved in 0.1% trifluoroacetic acid (TFA) and spotted in a HCCA (R-cyano-4-hydroxycinnamic acid) matrix (Sigma-Aldrich). The mass spectra were recorded in the 500–5000 Da range, using a minimum of 150 shots of laser per spectrum. Internal calibration was performed using trypsin autolysis fragments (*m*/*z* 842.5100, 1045.5642, and 2211.1046 Da). Peptide mass fingerprinting was compared to the theoretical masses from the Swiss-Prot databases using the Mascot algorithms [17]. Search parameters were: 50 ppm of mass tolerance, carbamidomethylation of cysteine residues, oxidation of methionine residues and one missed enzymatic cleavage for trypsin. A minimum of four peptide mass hits was required for a match.

Protein-protein interactions were deduced by the informatic platform of the STRING database [18].

## 3. Results

### 3.1. Activity Levels of MMP-2 and MMP-9 in Breast Cancer Tissues and Their Adjacent Non-Tumoral Tissues

Firstly, we investigated the collagenolytic activities in tumor tissues compared to their healthy counterparts adjacent to the tumor. Supplementary Table S1 reports the current clinical parameters of the selected patients.

**Figure 1:**
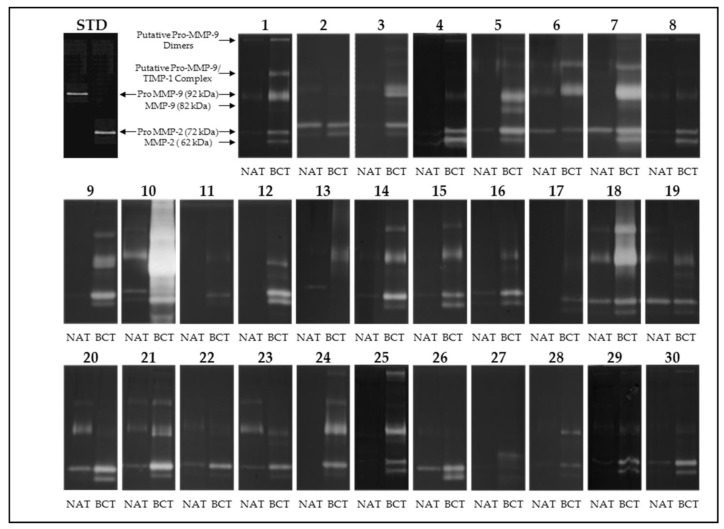
Gelatin zymography of 30 selected breast cancer tissues (BCT) and their non-tumoral adjacent tissues (NAT). Samples were run in parallel with purified MMP-2 and MMP-9 as a standard (lanes STD). The higher molecular weight (MW) of the lytic bands were previously identified as MMP-9 dimers and MMP-9/TIMP-1 complexes [14].

For this purpose, we performed gelatin zymographies in 30 pairs of tumoral and non-tumoral tissues run in parallel with purified MMP-2 and MMP-9 as a standard. As shown in Figure 1, the majority of the tumoral tissues, contrary to their non-tumoral counterparts, were positive for the lytic activities corresponding to the latent and activated form of MMP-2 (72 kDa and 62 kDa, respectively) and the latent and activated form of MMP-9 (92 kDa and 82 kDa, respectively). Occasionally, lytic bands of higher molecular weight also occurred in some patients. These bands may represent homodimeric forms of MMP-9 and complexes of pro-MMP-9/TIMP-1, respectively [14].

**Figure 2:**
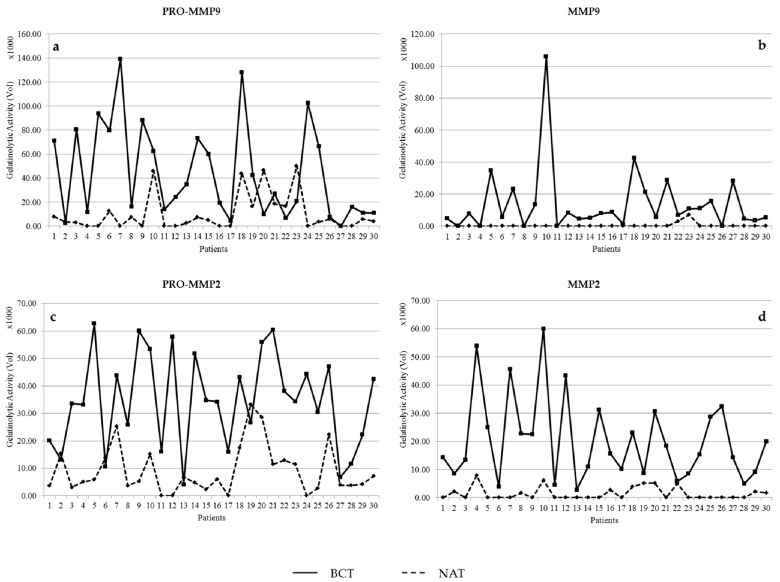
Densitometric profiles of the gelatinolytic bands corresponding to pro-MMP-9 (**a**); MMP-9 (**b**); pro-MMP-2 (**c**); MMP-2 (**d**) in the 30 breast cancer tissues compared to the respective non-tumoral adjacent tissues. Note that the active forms of both the MMPs are absent or rarely present in the non-tumoral tissues.

Gelatin zymograms were subjected to densitometric analysis to quantify the activity levels as relative volumes, using the Image Master software. The activity levels of pro-MMP-2, MMP-2, pro-MMP-9, and MMP-9 in tumor tissues were compared with the corresponding activities of the adjacent non-tumoral tissues when positive for lytic activity. As shown in the graphs in Figure 2, the activity levels of MMP-2 and MMP-9 were much higher in the tumoral tissues, compared to the positive non-tumoral tissues.

### 3.2. Gelatinolytic Activities of MMP-2 and MMP-9 in Breast Cancer Patients

We further extended the analysis to 80 BC surgical tissues to evaluate the activity levels of gelatinases in a significant number of cases. Supplementary Table S2 reports the current clinical parameters of the selected patients.

**Figure 3:**
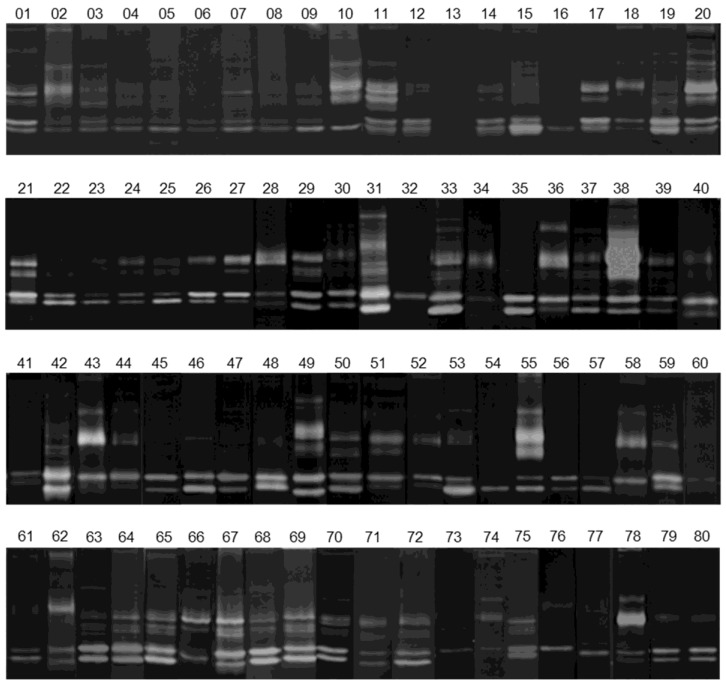
Gelatin zymographies of tissue extratcs from 80 breast cancer patients, showing a heterogeous level of the lytic bands. Gels were loaded with 18 ug of protein extracts and developed overnight at 37 °C, before staining with Coomassie-blue. Note the heterogeneous intensity level of the lytic bands among the patients.

**Figure 4:**
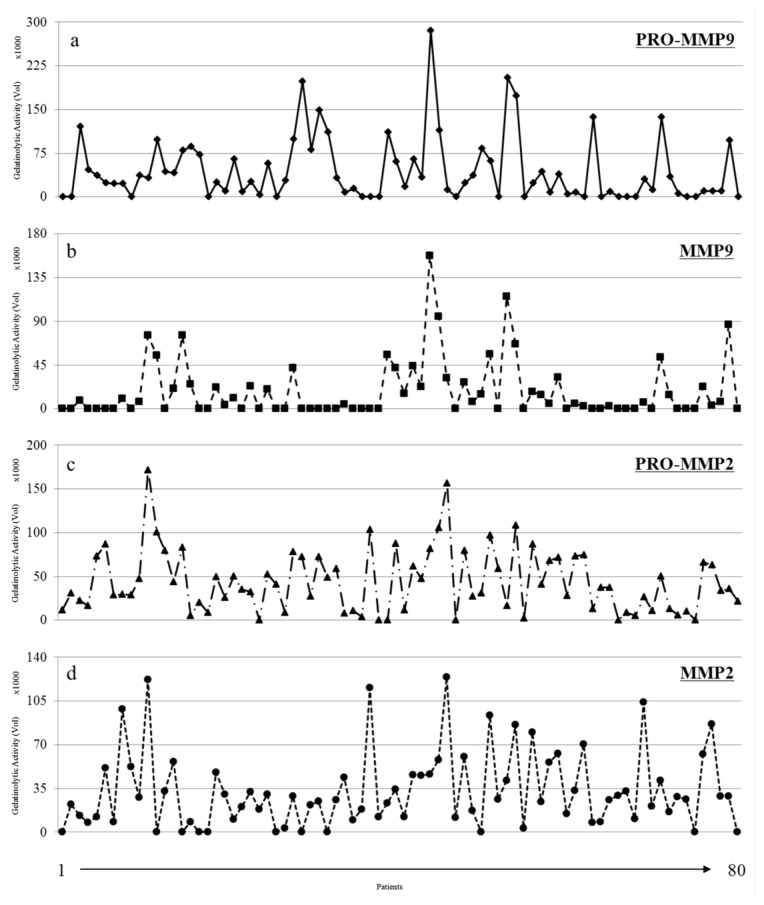
Densitometric profiles of the gelatinolytic bands identified as pro-MMP-9 (**a**), MMP-9 (**b**), pro-MMP-2 (**c**), MMP-2 (**d**) in the 80 breast cancer tissues.

As shown in Figure 3, the gelatinolytic activity was detected in almost all breast cancer tissues analyzed, even though some variations concerning the presence and intensity levels of the zymographic bands were seen. The diagrams in Figure 4 also show this heterogeneity among the selected cases.

In addition, we compared the activity levels of the collagenolytic bands within the group of 80 patients in order to find possible cross-correlations between them. The volumes derived from the densitometric analyses of each lytic band, as described in “Materials and Methods”, were used for the measurement of the Pearson’s correlation coefficient (*r*) by using the “Graphpad” system (PRISM4 Demo software).

The results showed a weak linear correlation between the pro-MMP2 and pro-MMP9, a significant correlation between the pro-enzymatic forms and their respective activated forms, and finally a moderate correlation between the two activated enzymes (Table 1).

### 3.3. MMP Levels and Proteomic Correlations

**Figure 5:**
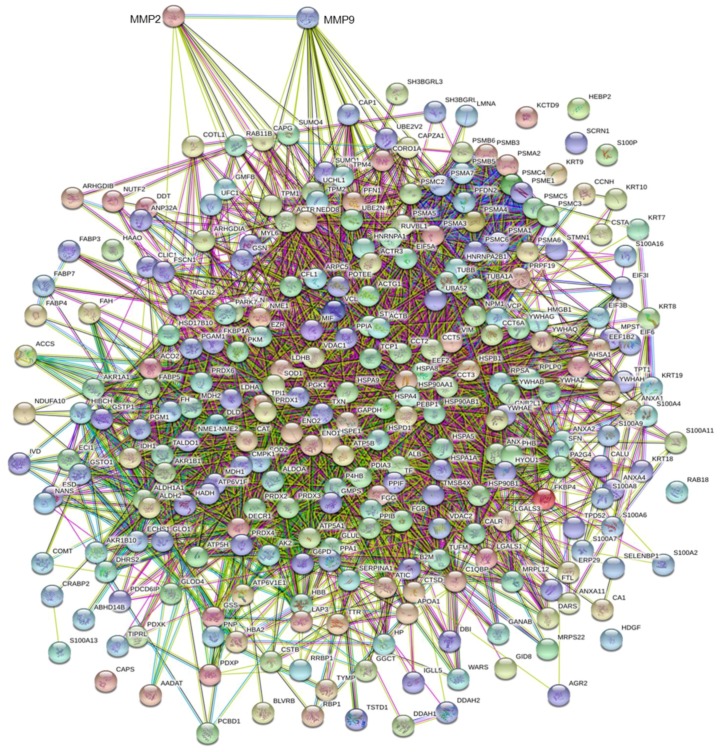
Interactome of the 274 genes coding for the 458 protein spots identified on the proteomic maps of the 80 breast cancer tissues selected for the study. The interactome was obtained by the software STRING, available online [18]. MMP-2 and MMP-9 were added to the list of 274 genes, in order to trace their predicted interactions with the proteomic profiles of the selected breast tissues.

**Figure 6:**
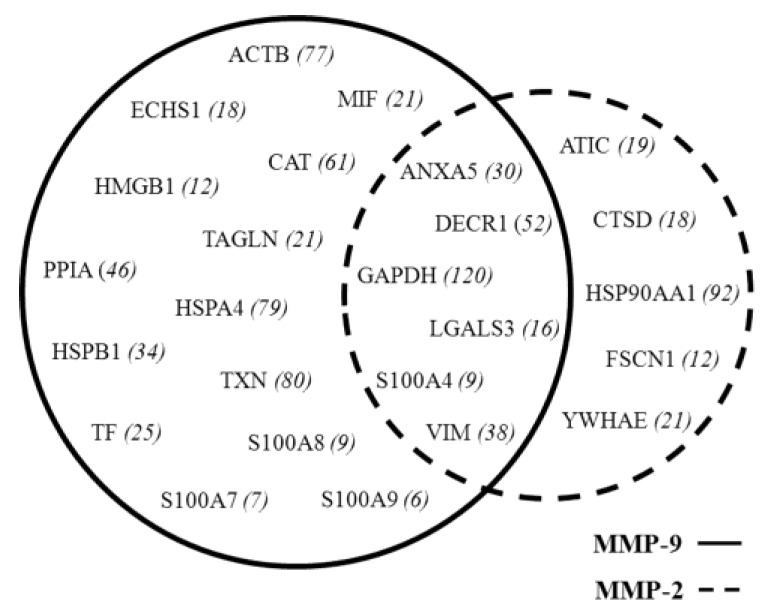
Venn diagram representing the predicted direct interactions of MMP-9 and MMP-2 with protein identities within the 274 entries identified in the proteomic profiles of the 80 selected breast cancer tissues. The intersection of the two circles includes six identities shared by the two MMPs. In brackets are the reported second-level interactors for each putative partner.

Surgical tissue fragments from the group of 80 selected patients were submitted for proteomic analysis according to the methodology described [15]. Collectively, the spots identified with UniProt Accession number (AC), were 458 proteins (including isoforms) corresponding to 274 AC entries. This list (see Supplementary Table S3) was subjected to bio-informatic analyses. Qualitative correlations were deduced by the “STRING” functional protein association networks, through known/predicted evidence. This platform traced the network between the tissue proteins of the tumoral samples with MMP-2 and MMP-9, respectively, as illustrated by the diagrams in Figure 5. In detail, by entering the list of the 274 AC identities with MMP-9, the system generated a network of interactions, 20 of which were directly linked, while many others occurred through other intermediate interactors. The same analysis with MMP-2 revealed 11 direct interactions. As shown in the Venn diagram in Figure 6, six interactors were common between the two enzymes. Each protein of the network can interplay with a variable number of secondary interactors, as reported in brackets.

Collectively, these have been classified into the following categories according to the current gene ontologies: cell proliferation, anti-apoptosis, metabolic pathways, cytoskeleton organization and cell motility, cell surface projections and response to stress and extracellular activities.

**Figure 7:**
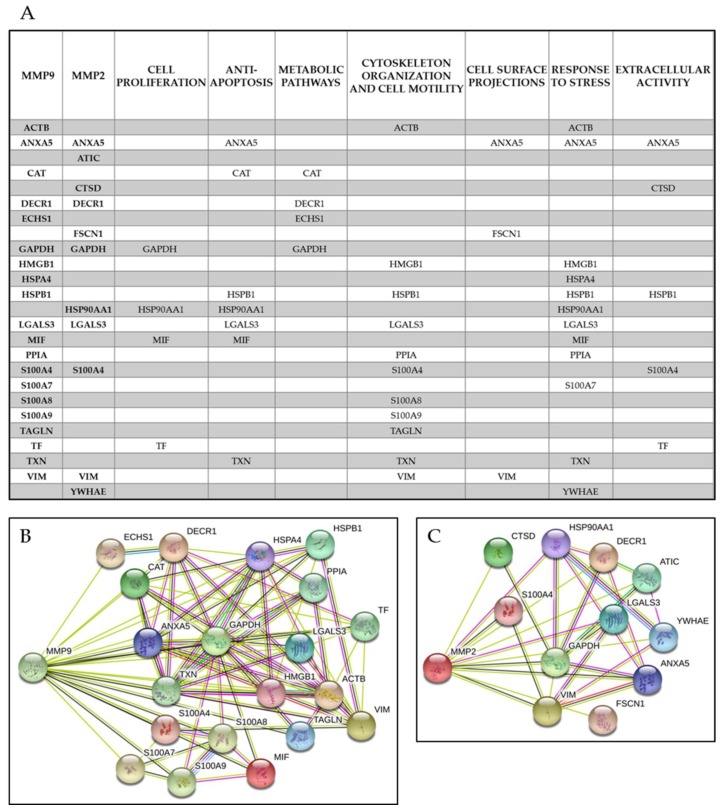
(**A**) Lists of the direct interactions of MMP-9 and MMP-2 with their putative partners at the primary level. The interacting proteins are distributed in the functional categories given by the databases. The lower panels show the interactors of MMP-9 (**B**) and MMP-2 (**C**), generated by and extracted from the STRING platform as shown in Figure 5.

Due to the multifunctional roles of the majority of the detected proteins, they were included in several functional classes, as shown in Figure 7a–c, which highlights the interactors of MMP-9 (b) and MMP-2 (c) generated by and extracted from the STRING platform (Figure 5).

## 4. Discussion

The potentially important role of matrix-degrading enzymes in breast cancer has been stated for many years, in particular in relation to the activities of MMP-2 and MMP-9 [14]. Indeed, both collagenolytic enzymes have been individually correlated with breast cancer progression [19] and tumor vascularization, invasion and metastasis, and differentiation and proliferation [20]. We initially aimed to verify the occurrence of gelatinase activity in 30 tissue samples of breast carcinomas vs. the adjacent non-affected tissues. The results demonstrated that both MMP-9 and MMP-2 were present, as pro- and active forms in the majority of the tumoral tissues, and absent or detectable at very low levels in the adjacent non-affected paired tissues.

This result encouraged us to continue investigating the expression of the lytic activities of the pro- and active forms of MMP-2 and MMP-9 on a larger number of cases, consisting of 80 surgical tissue fragments.

These two collagenolytic enzymes have been traditionally considered to be the most important promoters of tumor invasion, because of their ability to degrade the first tissue barrier, i.e., the basal lamina. This, as is well known, has the role of maintaining the right tissue architecture and to prevent cell mobility of stationary tissues, such as epithelial tissue.

The first question of our approach was to understand if the two enzymatic activities were interrelated. The results showed a significant Pearson correlation between the pro-enzymatic forms and their corresponding activated forms; while a moderate correlation was found between the two active enzymes. These observations suggest that the two enzymes can act in an independent manner, but in some way complementary.

In the belief that genes and proteins never operate alone, we wanted to search for potential interactors of the two enzymes. For this purpose, we used the STRING platform that builds functional protein association networks based on literature evidence.

An impressive result was the observation of a noticeably high number of hypothetical functional interactions at several levels. The first level concerns the primary interactions that each enzyme can establish with its putative partners. The second level concerns the secondary interactions, and beyond, and presumes the presence of intermediaries among the functional interactions.

### 4.1. Common Interactors for MMP-2 And MMP-9

The first-level interactions were 20 contacts for MMP-9, and 11 for MMP-2. A point of interest was the observation that six contacts are shared between the two enzymes, namely: GAPDH, ANXA5, LGAL3, S100A4, DECR1 and VIM.

*Glyceraldehyde-3-phosphate dehydrogenase, G3P (GAPDH).* Among the shared interactors, the presence of the G3P, which is generally over-expressed in breast cancer, as already reported in our previous studies [21], deserves particular attention. The G3P, besides being a key enzyme of glycolysis, under some circumstances, can translocate into the nucleus and act as a co-promoter for cell proliferation [22,23]. Its putative relationship with the MMPs has never reported before.

*Annexin A5, ANXA5 (ANX5A).* This protein is a member of a multigene family consisting of 13 members of Ca^2+^ and phospholipid binding proteins with peripheral membrane location. In our previous proteomics study, we found a ubiquitous and over-expressed presence of ANXA5, which is known to play an anti-apoptotic role. Similarly to other members of the family, it is thought to be involved at different levels in the tumor development, progression and invasivity [24,25,26]. Moreover, Annexin A5 is known to exert an anticoagulant effect, acting as an indirect inhibitor of the thromboplastin-specific complex [27].

*Galectin, LEG3 (LGALS3)*. Galectin is a galactose-specific lectin associated with the cell membrane and involved in several processes of membrane trafficking by interacting with other surface proteins [28]. Intracellularly, it participates in the cytoskeleton organization and cell motility by interacting with ACTB and other associated proteins (COF1, EZRI, MIF, and CAPG). Extracellularly, it interacts with a variety of cell surface glycoproteins, and in cancer, it seems to participate in the dynamics of cell migration and in escaping the T cell-mediated immune response [29].

*S100 proteins.* The S100 proteins represent a multigene family of calcium-binding proteins of the EF-hand type, encoded by 21 genes in humans [30]. Members of the S100 family are differentially expressed in normal tissues and are frequently upregulated in cancer [31]. They may perform a large variety of functions, either intracellularly or extracellularly, in a cytokine-like manner through the receptor for “advanced glycation end products” (RAGE) [32]. In particular, the S100A4 protein is known to be secreted by tumor and/or stromal cells to support tumorigenesis by stimulating angiogenesis and promoting endothelial cell migration [33].

In addition, it has been reported that S100A4 may support MMP-9 and MMP-13 gene expression [34], and also that it may enhance the activity of some MMPs, causing higher cell dissociation and cancer metastasis [35,36].

*Vimentin, VIME (VIM).* Another interesting partner of the MMPs appears to be VIM. Vimentins are class-III intermediate filaments found in various non-epithelial cells, especially mesenchymal cells. Its high expression in breast cancer testifies to the occurrence of the epithelial-mesenchyme transition, already postulated at the proteomic level [21]. Its partnership with MMP-2 and -9 is suggestive for joined mechanisms of matrix degradation and cell migration.

*2,4-Dienoyl-CoA reductase, DECR (DECR1)*. Somewhat ambiguous is the involvement of DECR1 2,4-dienoyl-CoA reductase, a mitochondrial auxiliary enzyme of beta-oxidation, for which no reports have been produced in relation to cancerogenesis, or with the MMPs in particular.

### 4.2. MMP-9 Direct Interactors

Fourteen out of the 20 putative interactors were exclusive for MMP-9. They were the following:

*Transgelin, TAGL (TAGLN)* is an actin-binding protein involved in calcium interactions and contractile properties of the cell. Using an expression cloning strategy, Nair et al. identified transgelin as a novel suppressor of MMP-9 expression [37]. This finding attributes the unusual role of tumor-suppressor to the MMP-9, which deserves further attention.

*Macrophage Migration Inhibitory Factor, MIF (MIF)* is an interesting small protein (approximately 12.5 kDa) involved in several biological activities, including the stimulation of the production of cytokines, chemokines, growth factors and angiogenic factors that may favor tumor growth and metastatic spreading. The overexpression of MIF in breast cancer cells, and its reported interaction with HSP90 and CXCR-4, is known to induce resistance to apoptosis and stimulation of proliferation via the AKT pathway. This opens new scenery regarding the possible correlations between matrix degradation and cell proliferation. Moreover, MIF is involved in the innate immune response and in regulating the function of macrophages in host defense [38,39,40].

*Heat Shock 70 kDa Protein 4, HSP74 (HSPA4)* and *Heat Shock 27kDa Protein, (HSP27) (HSPB1)* are two significant members of the multigenic heat shock protein family [41]. The HSPA4 (heat shock 70kDa protein 4) is a major component of the HSP chaperone family involved in the folding of nascent proteins and in the degradation of misfolded polypeptides [42].

The other subset of chaperones consists of HSPs of a molecular weight of less than 30 kDa (sHSPs). Among some of their functions, sHPSs participate in cell survival, cytoskeletal motility, and disruption of protein aggregation [43]. Moreover, it has been reported that the HSPs display elevated expression levels in cancer, where they may perform anti-apoptotic activities, both spontaneous and generated by therapy [44]. In particular, the high expression of HSPB1 (HSP27) has been associated with poor prognosis in several carcinomas and osteosarcomas [45]. Their putative correlation with MMP-9 suggests a synergy between the two key mechanisms of cancer progression: matrix degradation and anti-apoptotic effects.

*Peptidylprolyl isomerase A (cyclophilin A), PPIA (PPIA)* accelerates the folding of proteins and catalyzes the cis-trans isomerization of proline imidic peptide bonds in oligopeptides. Recently, a key role of PPIA in tumor biology has been proposed [46]. The postulated interaction with MMP-9 remains to be clarified.

Other presumed interactors are listed below.

*Catalase, CATA (CAT)* occurs in almost all aerobic organisms and serves to protect cells from the toxic effects of hydrogen peroxide (H_2_O_2_). Moreover, it can reduce the activity of MMPs and promote cellular growth of many cell types, including T-cells, B-cells, myeloid leukemia cells, transformed fibroblast cells and others [47,48].

*Enoyl-CoA hydratase, ECHM (ECHS1)* is a key enzyme involved in the oxidation of fatty acids and branched-chain amino acids. Moreover, it has been reported to be associated with the progression of a variety of tumors [49], including gastric cancer [50]. However, no correlation has been described so far with the matrix metalloproteases.

*Thioredoxin, THIO (TXN)* is a multifunctional cellular factor with thiol-mediated redox activity. It plays pivotal roles in the regulation of many cellular processes, including proliferation, apoptosis, and gene expression, both in normal and tumoral cells [44]. Its frequent high expression has been reported in many cancers [51,52,53].

*Transferrin, TRFE (TF)* is a transport protein which can bind two Fe (3+) ions in association with the binding of an anion, usually bicarbonate. It is responsible for the transport of iron from sites of absorption and heme degradation to those of storage and utilization. Serum transferrin may also have a further role in stimulating cell proliferation [54].

*High mobility group protein B1, HMGB1 (HMGB1)* is a multifunctional redox-sensitive protein with various roles in different cellular compartments. In the nucleus, it is one of the major non-histone/chromatin-associated proteins and acts as a DNA chaperone involved in replication, transcription, DNA repair and genome stability. It has been reported that it promotes host inflammatory reactions to external signals and immune responses. In the cytoplasm, it functions as a damage-associated molecule inducing inflammatory mediator release [55].

*S100 proteins*, such as the group of S100-A7,-A8 and -A9 calcium binding proteins, are known to exert several functions in normal and tumoral tissues, where they may be expressed at high levels (as has been reported by our group [56] and other authors [57,58,59,60]). Their correlation with MMP-9 has never been proposed before.

*Actin, (ACTB) ACTB* is the primary component of the cytoskeleton in eukaryotic cells. The actin cytoskeleton is therefore responsible for the integrity and shape-maintenance of cells. The cytoskeleton reorganization is a physiological event during cell growth, differentiation, and senescence of stationary cells, and it is responsible for the cell motility of migratory cells [61].

Under pathological conditions, like cancer, the actin cytoskeleton may undergo deregulated fragmentation. It has been recently reported that induced alteration of actin cytoskeletal integrity in human trabecular meshwork cells (HTMC) is associated with MMP-2 activation, presumably through the upregulation of its activator, MT1-MMP [62].

### 4.3. MMP-2 Direct Interactors

The functional interactors attributed exclusively to MMP-2 are the following:

*Bifunctional purine biosynthesis protein PURH, PUR9 (ATIC)* is a bifunctional enzyme that catalyzes two steps in purine biosynthesis. Moreover, it has been reported that it may promote auto-phosphorylation and internalization of the insulin receptor (INSR) [63]. No information is currently found on potential functional interactions with MMP-2 and other MMPs

*Cathepsin D, CATD (CTSD)* is an acid protease that is active in the breakdown of intracellular proteins, which has been reported to be involved in the pathogenesis of several diseases, such as breast cancer and possibly Alzheimer’s disease via non-proteolytic pathways [64,65]. The postulated direct correlation between CTSD and MMP-2 is an interesting observation which needs further insight.

*Fascin, FSCN1 (FSCN1)* is one of the organizers of the actin filaments into bundles. Therefore, it plays a role in the formation of microspikes, membrane ruffles, and stress fibers, as well as other cell projections such as filopodia, which are essential for cell motility and migration. Its frequent overexpression in cancer has been related to the promotion of an actin-independent cell migration [66]. Its correlation with MMP-2 could be instrumental in the increased aptitude of cells to migrate during the invasive phase of the breast cancer,

*Heat shock protein HSP 90-alpha, HS90A (HSP90AA1)* is a molecular chaperone that has been recognized as one of those responsible for the structural maintenance of cells, and also for cell cycle control and signal transduction pathways [67]. It has been reported that HSP90, when translocated into the nucleus, may influence the activity of many transcription factors [68].

*14-3-3 Protein epsilon, 1433E (YWHAE)* is a member of a multigenic protein family. In mammals, it consists of seven members (β, ε, η, γ, τ, ζ and σ), which act as adapter proteins involved in the regulation of a large number of signaling pathways [69,70] and in the maintenance of epithelial cell polarity [71]. It is interesting to note that this protein is frequently overexpressed in breast cancer [21]. However, no correlation with collagenolytic activities has been reported before.

In conclusion, the present investigation (based on a double line of approach, experimental and “in silico”) highlights for the first time the complexity of the interactive networks that MMP-2 and MMP-9, through a series of interactors (partly common and partly exclusive) may accomplish within the cell. These complex interactive molecular circuits, where the two collagenolytic enzymes appear to be included, suggest their potential involvement in other important cellular activities, besides that of remodeling the extracellular matrix. These interactors are deduced by the direct inquiry between MMP-2 and MMP-9 with the proteomic platform of the breast tissues, and are not inclusive of the MMP inhibitors (TIMP) and activator (MMP14). The scenery becomes even more complex when analyzing the members of the second level of interactors, or beyond. In our opinion, this is the first comprehensive description of potential activities where MMP-2 and MMP-9 can be involved in the complicated scenario in which the mechanisms of tumor progression are correlated with unfavorable prognosis.

## Figures and Tables

**Table 1:** Statistical correlations (Pearson *r*) between the activity levels of pro-MMP-9/pro-MMP-2; pro-MMP-9/MMP-9; pro-MMP-2/MMP-2 and MMP-9/MMP-2, detected in the breast cancer extracts from the 80 patients selected for this study. The number of asterisks indicates the level of significance.

	PRO-MMP-9/PRO-MMP-2	PRO-MMP-9/MMP-9	PRO-MMP-2/MMP-2	MMP-9/MMP-2
Number of XY Pairs	80	80	80	80
Pearson *r*	0.249	0.6805	0.6244	0.292
95% confidence interval	0.03091 to 0.4444	0.5418 to 0.7831	0.4690 to 0.7423	0.07722 to 0.4809
*p* value (two-tailed)	0.0259	*p* < 0.0001	*p* < 0.0001	0.0086
*p* value summary	*	***	***	**
Is the correlation significant? (alpha = 0.05)	Yes	Yes	Yes	Yes
R squared	0.06199	0.4631	0.3899	0.08529

## Supplementary Materials

The following are available online at http://www.mdpi.com/2073-4409/7/8/89/s1, Table S1: Basic clinical parameters corresponding to 30 selected patients, Table S2: Basic clinical parameters corresponding to 80 selected patients, Table S3: List of 274 genes encoding for 458 protein spots identified on our reference proteomic map of Breast Cancer Tissue.
