# Supplementary material for: New Insights into the Occurrence of Matrix Metalloproteases -2 and -9 in a Cohort of Breast Cancer Patients and Proteomic Correlations
Source: Cells. 2018 Jul 28;7(8):89. doi: 10.3390/cells7080089 (PMC6115737; doi:10.3390/cells7080089)
Supplement: Supplementary file 1 [file cells-07-00089-s001.pdf]

Supplementary Table S1. Basic clinical parameters corresponding to the 30 patients selected for the zymographic assay shown in Figure 1. T: tumor size; N: lymph node status; ER: Estrogen Receptors; PGR: Progesteron Receptors; G: tumor grading.

Zymographic Lane	T	N	HER2	ER%	PGR%	Ki67 %	G
1	2	3	3+	90	60	50	G3
2	2	0	n.d.	8	2	n.d.	G2
3	1c	NX	n.d.	5	7	n.d.	G2
4	2	2a	neg.	90	40	60	G3
5	3	3a	neg.	10	neg.	n.d.	G3
6	2	NX	neg.	35	8	n.d.	G2
7	1	2	neg.	6	9	n.d.	G2
8	2	1a	neg.	80	90	35	G3
9	3	NX	neg.	50	40	n.d.	G3
10	3	NX	2+	90	80	n.d.	G3
11	3	1b	n.d.	13	18	n.d.	G2/G3
12	2	1b	3+	60	neg.	n.d.	G3
13	1	0	n.d.	<1	1	n.d.	G2
14	2	0	n.d.	13	7	n.d.	G2
15	1c	0	2+	1	13	n.d.	G2
16	1c	1	3+	3	6	n.d.	G2
17	2	1	neg.	20	10	50	G2
18	1c	3a	neg.	neg.	neg.	40	G3
19	2	0	neg.	neg.	neg.	n.d.	G3
20	2	2a	neg.	90	40	60	G3
21	2	0	neg.	80	90	n.d.	G2
22	TX	NX	3+	neg.	neg.	n.d.	G3
23	2	1a	neg	80	90	n.d.	G3
24	2	NX	neg.	neg.	neg.	n.d.	G3
25	3	2	neg.	80	80	10	G2
26	2	NX	n.d.	3	2	n.d.	G2
27	1c	0	neg.	80	80	5	G3
28	2	NX	2+	neg.	neg.	n.d.	G3
29	2	1b	2+	80	<5	35	G3
30	4	1	neg.	neg.	neg.	10	G3

Supplementary Table S2. Basic clinical parameters corresponding to the 80 patients selected for the zymographic assay shown in Figure 3. T: tumor size; N: lymph node status; ER: Estrogen Receptors; PGR: Progesteron Receptors; G: tumor grading.

Zymographic Lane	T	N	HER2	ER%	PGR%	Ki67 %	G
1	2	1	2+	neg.	neg.	40	G3
2	1c	0	2+	95	10	30	G2
3	2	0	neg.	95	30	70	G3
4	2	1a	neg.	90	65	14	G2
5	2	2a	3+	65	5	25	G2
6	2	2a	neg.	95	60	70	G3
7	2	0	neg.	95	80	20	G2
8	2	1a	neg.	95	90	50	G3
9	2	1	neg.	85	2	17	G2
10	2	0	neg.	3	neg.	61	G3
11	1c	0	neg.	10	neg.	40	G3
12	2	2	neg.	70	80	40	G2/G3
13	2c	1	neg.	80	70	9	G2
14	2	0	3+	70	70	25	G2
15	2	1a	2+	95	90	15	G2
16	1c	1	neg.	95	80	25	G2
17	2	3	3+	neg.	5	50	n.d.
18	TX	NX	n.d.	n.d.	n.d.	n.d.	G3
19	2	2a	neg.	95	95	36	G3
20	2	0	neg.	neg.	neg.	60	G3
21	2	1b	3+	70	60	20	G3
22	2	0	2+	90	40	35	G2
23	3	2	neg.	80	0-1	16-18	G3
24	2	3	neg.	90	neg.	15	G3
25	1a	0	neg.	70	75	2	G2
26	2	2	neg.	80	40	n.d.	G3
27	2	1	2+	80	5	60	G3
28	1c	NX	n.d.	5	7	n.d.	G2
29	2	0	neg.	neg.	neg.	60	G3
30	2	1	2+	95	80	15	G1/G2
31	4	3	2+	neg.	neg.	85	G3
32	2	1a	neg	80	90	n.d.	G3
33	2	2a	3+	70	60	45	G3
34	1	0	2+	70	60	18	G2
35	1c	1	neg.	95	95	7	G1/G2
36	TX	NX	neg	50	40	n.d.	G3
37	2	0	neg.	85	85	60	G3
38	1c	2	3+	75	60	50	G3

Supplementary Table S2. *Cont.*

Zymographic Lane	T	N	HER2	ER%	PGR%	Ki67 %	G
39	1c	0	neg.	neg.	neg.	70	G3
40	1c	1	3+	3	6	n.d.	G2
41	2	0	n.d.	8	2	n.d.	G2
42	1c	0	neg.	95	40	32	G3
43	3	0	neg.	neg.	neg.	0	G3
44	TX	NX	3+	neg.	neg.	n.d.	G3
45	1c	1	neg.	40	60	45	G2
46	2	0	neg.	95	95	22	G1/G2
47	1c	0	neg.	95	20	40	G3
48	1c	0	neg.	80	80	5	G3
49	1c	0	neg.	neg.	neg.	60	G3
50	1c	1a	neg.	95	3	25	G2
51	2	3a	neg	10	0	n.d.	G3
52	1c	0	2+	1	13	n.d.	G2
53	1c	0	neg.	neg.	neg.	28	G3
54	2	3	neg.	neg.	neg.	65	G3
55	2	1a	neg.	neg.	neg.	85	G3
56	1c	0	neg.	neg.	neg.	10	G3/G2
57	2	1a	neg.	neg.	neg.	25	G3
58	2	NX	neg.	neg.	neg.	n.d.	G3
59	2	1b	3+	60	neg.	n.d.	G3
60	3	1b	n.d.	13	18	n.d.	G2/G3
61	2	1a	neg.	95	95	25	G2
62	0	0	neg.	neg.	neg.	20	G3
63	1c	0	neg.	80	60	20	G2
64	2	1	neg.	95	90	22	G2
65	1c	1s	neg.	60	70	5	G1/G2
66	2	0	neg.	80	30	35	G2
67	2	2a	neg.	95	95	60	G3
68	2	1a	neg.	65	40	12	G1/G2
69	2	1a	3+	65	85	45	G3
70	2	3	3+	neg.	neg.	35	G3
71	3	2a	neg.	neg.	neg.	90	G3
72	2	0	2+	75	65	85	G2
73	1c	1	3+	80	5	30	G3
74	2	0	neg.	80	80	18	G2
75	1c	1	2+	70	70	25	G2
76	2	2	neg.	90	85	20	G2/G3
77	3	1	neg.	neg.	neg.	28	G3
78	2	2	neg.	70	80	10	G3

Supplementary Table S2. *Cont.*

Zymographic Lane	T	N	HER2	ER%	PGR%	Ki67 %	G
79	1c	2a	3+	neg.	neg.	30	G3
80	2	2	neg.	85	25	40	G3

Supplementary Table S3. List of the 274 genes encoding for the 458 protein spots identified on our reference proteomic map of Breast Cancer Tissue. The UniProt Accession Number (AC) corresponds to the access number of the Swiss-Prot/TrEMBL database.

Gene Name	UniProt AC
AADAT	Q8N5Z0
ABHD14B	Q96IU4
ACCS	Q96QU6
ACO2	Q99798
ACTB	P60709
ACTG1	P63261
ACTR1B	P42025
ACTR3	P61158
AGR2	O95994
AHSA1	O95433
AK2	P54819
AKR1A1	P14550
AKR1B1	P15121
AKR1B10	O60218
ALB	P02768
ALDH1A1	P00352
ALDH2	P05091
ALDOA	P04075
ANP32A	P39687
ANXA1	P04083
ANXA11	P50995
ANXA2	P07355
ANXA4	P09525
ANXA5	P08758
APOA1	P02647
ARHGDIA	P52565
ARHGDIB	P52566
ARPC5	O15511
ATIC	P31939
ATP5A1	P25705
ATP5B	P06576
ATP5H	O75947
ATP6V1E1	P36543
ATP6V1F	Q16864
B2M	P61769
BLVRB	P30043

Supplementary Table S3. *Cont.*

Gene Name	UniProt AC
C1QBP	Q07021
CA1	P00915
CALR	P27797
CALU	O43852
CAP1	Q01518
CAPG	P40121
CAPS	Q13938
CAPZA1	P52907
CAT	P04040
CCNH	P51946
CCT2	P78371
CCT3	P49368
CCT5	P48643
CCT6A	P40227
CFL1	P23528
CLIC1	O00299
CMPK1	P30085
COMT	P21964
CORO1A	P31146
COTL1	Q14019
CRABP2	P29373
CSTA	P01040
CSTB	P04080
CTSD	P07339
DARS	P14868
DBI	P07108
DDAH1	O94760
DDAH2	O95865
DDT	P30046
DECR1	Q16698
DHRS2	Q13268
DLD	P09622
ECHS1	P30084
ECI1	P42126
EEF1B2	P24534
EEF2	P13639
EIF3B	P55884
EIF3I	Q13347

Supplementary Table S3. *Cont.*

Gene Name	UniProt AC
EIF5A	P63241
EIF6	P56537
ENO1	P06733
ENO2	P09104
ERP29	P30040
ESD	P10768
EZR	P15311
FABP3	P05413
FABP4	P15090
FABP5	Q01469
FABP7	O15540
FAH	P16930
FGB	P02675
FGG	P02679
FH	P07954
FKBP1A	P62942
FKBP4	Q02790
FSCN1	Q16658
FTL	P02792
G6PD	P11413
GANAB	Q14697
GAPDH	P04406
GGCT	O75223
GID8	Q9NWU2
GLO1	Q04760
GLOD4	Q9HC38
GLUL	P15104
GMFB	P60983
GMPS	P49915
GSN	P06396
GSS	P48637
GSTO1	P78417
GSTP1	P09211
HAAO	P46952
HADH	Q16836
HBA1	P69905
HBA2	P69905
HBB	P68871

Supplementary Table S3. *Cont.*

Gene Name	UniProt AC
HDGF	P51858
HEBP2	Q9Y5Z4
HIBCH	Q6NVY1
HMGB1	P09429
HNRNPA1	P09651
HNRNPA2B1	P22626
HP	P00738
HSD17B10	Q99714
HSP90AA1	P07900
HSP90AB1	P08238
HSP90B1	P14625
HSPA1A	P0DMV8
HSPA4	P34932
HSPA5	P11021
HSPA8	P11142
HSPA9	P38646
HSPB1	P04792
HSPD1	P10809
HSPE1	P61604
HYOU1	Q9Y4L1
IDH1	O75874
IGHG1	P01857
IGLC1	P0CG04
IVD	P26440
KCTD9	Q7L273
KRT10	P13645
KRT18	P05783
KRT19	P08727
KRT7	P08729
KRT8	P05787
KRT9	P35527
LAP3	P28838
LDHA	P00338
LDHB	P07195
LGALS1	P09382
LGALS3	P17931
LMNA	P02545
MDH1	P40925

Supplementary Table S3. *Cont.*

Gene Name	UniProt AC
MDH2	P40926
MIF	P14174
MPST	P25325
MRPL12	P52815
MRPS22	P82650
MYL6	P60660
NANS	Q9NR45
NDUFA10	O95299
NEDD8	Q15843
NME1	P15531
NME2	P22392
NPM1	P06748
NUTF2	P61970
P4HB	P07237
PA2G4	Q9UQ80
PARK7	Q99497
PCBD1	P61457
PDCD6IP	Q8WUM4
PDIA3	P30101
PDXK	O00764
PDXP	Q96GD0
PEBP1	P30086
PFDN2	Q9UHV9
PFN1	P07737
PGAM1	P18669
PGK1	P00558
PGM1	P36871
PHB	P35232
PKM	P14618
PNP	P00491
POTEE	Q6S8J3
PPA1	Q15181
PPIA	P62937
PPIB	P23284
PPIF	P30405
PRDX1	Q06830
PRDX2	P32119
PRDX3	P30048

Supplementary Table S3. *Cont.*

Gene Name	UniProt AC
PRDX4	Q13162
PRDX6	P30041
PRPF19	Q9UMS4
PSMA1	P25786
PSMA2	P25787
PSMA3	P25788
PSMA4	P25789
PSMA5	P28066
PSMA6	P60900
PSMA7	O14818
PSMB3	P49720
PSMB5	P28074
PSMB6	P28072
PSMC2	P35998
PSMC3	P17980
PSMC4	P43686
PSMC5	P62195
PSMC6	P62333
PSME1	Q06323
RAB11B	Q15907
RAB18	Q9NP72
RACK1	P63244
RBP1	P09455
RPLP0	P05388
RPSA	P08865
RRBP1	Q9P2E9
RUVBL1	Q9Y265
S100A11	P31949
S100A13	Q99584
S100A16	Q96FQ6
S100A2	P29034
S100A4	P26447
S100A6	P06703
S100A7	P31151
S100A8	P05109
S100A9	P06702
S100P	P25815
SCRN1	Q12765

Supplementary Table S3. *Cont.*

Gene Name	UniProt AC
SELENBP1	Q13228
SERPINA1	P01009
SFN	P31947
SH3BGRL	O75368
SH3BGRL3	Q9H299
SOD1	P00441
SOD2	P04179
STIP1	P31948
STMN1	P16949
SUMO1	P63165
SUMO4	Q6EEV6
TAGLN	Q01995
TAGLN2	P37802
TALDO1	P37837
TCP1	P17987
TF	P02787
TIPRL	O75663
TMSB4X	P62328
TPD52	P55327
TPI1	P60174
TPM1	P09493
TPM2	P07951
TPM4	P67936
TPT1	P13693
TSTD1	Q8NFU3
TTR	P02766
TUBA1A	Q71U36
TUBB	P07437
TUFM	P49411
TXN	P10599
TYMP	P19971
UBA52	P62987
UBE2N	P61088
UBE2V2	Q15819
UCHL1	P09936
UFC1	Q9Y3C8
VCL	P18206
VCP	P55072

Supplementary Table S3. *Cont.*

Gene Name	UniProt AC
VDAC1	P21796
VDAC2	P45880
VIM	P08670
WARS	P23381
YWHAB	P31946
YWHAE	P62258
YWHAG	P61981
YWHAH	Q04917
YWHAQ	P27348
YWHAZ	P63104
